# Exogenous melatonin treatment on post-harvest jujube fruits maintains physicochemical qualities during extended cold storage

**DOI:** 10.7717/peerj.14155

**Published:** 2022-10-14

**Authors:** Yang Wang, Jirui Zhang, Qiaoli Ma, Xaio’ai Zhang, Xian Luo, Qunxian Deng

**Affiliations:** College of Horticulture, Sichuan Agricultural University, Chengdu, Sichuan, China

**Keywords:** Melatonin, Postharvest jujube, Visual appearance, Physicochemical changes

## Abstract

This study was conducted to investigate the visual appearance and physicochemical changes of postharvest jujube fruits (*Ziziphus jujuba* Mill. cv. Shucuizao) stored under 0 °C for 15 days. The fruits were dipped in 0 (control), 50, 100, 200 and 400 µM melatonin solutions after harvest. The results showed that treatment with a suitable melatonin concentration improved the rate of crisp fine fruits, delayed weight loss and firmness decline, and suppressed changes in total soluble solids (TSS) and titratable acidity (TA) contents of jujube fruits compared with the control. In addition, jujube fruits soaked with melatonin showed improved antioxidant capacity through increased ascorbic acid (AsA) content, enhanced superoxide dismutase (SOD) activity, and decreased malonaldehyde (MDA) content. As a result, 50 µM melatonin showed the greatest improvement of visual appearance and quality maintenance, and could be used as an effective treatment to preserve postharvest jujube fruit.

## Introduction

Melatonin, chemically named *N*-acetyl-5-methoxytryptamine, is a small indole molecule that exists in most biological organisms (Arnao & Hernández-Ruiz, 2015). Melatonin plays an important role in biological processes, such as plant growth (Nawaz et al., 2021), fruit ripening and senescence (Wang et al., 2020), and biotic and abiotic stress responses (Tiwari et al., 2020). Growing evidence shows that exogenous melatonin had a positive effect on the preservation of postharvest fruits (Bal, 2019). Melatonin treatment attenuated postharvest decay of strawberry (Liu et al., 2018; Aghdam & Fard, 2017), litchi (Zhang et al., 2021) and peach (Gao et al., 2016), improved the antioxidant capacity of loquat (Wang et al., 2021a; Wang et al., 2021b) and sweet cherry (Sharafi et al., 2021), and maintained the quality of orange (Ma et al., 2021) during cold storage, thus extending postharvest life of fresh fruits.

Jujube (*Ziziphus jujuba* Mill.) is a characteristic economic forest fruit native to China (Liu et al., 2014). Jujube fruit is very popular among consumers due to its unique aroma and taste, as well as its high content of ascorbic acid (AsA), phenolic compounds, triterpene acids, mineral elements, and other medicinal and edible homologous substances (Bai et al., 2016; Guo et al., 2015; Islam et al., 2022; Karakaya et al., 2020; Ozturk et al., 2021; Pu et al., 2017; Tahergorabi et al., 2015). Postharvest jujube is highly perishable due to its strong respiration intensity and metabolism. When stored at room temperature and humidity for 3–5 d, jujube fruit could lose water, shrink, soften and brown, which has an adverse effect on transportation and sales, and seriously reduces its economic value (Liu & Wang, 2019; Shams Najafabadi et al., 2021; Yu et al., 2021).

Exogenous melatonin has also been used in the storage and preservation of jujube. However, the optimal melatonin concentration for preservation of postharvest fresh jujube could differ due to differences of variety, storage temperature and treatment method (Deng et al., 2021; Wang et al., 2021a; Wang et al., 2021b; Tang et al., 2020). Jujube cv. Shucuizao is an excellent fresh variety with high fruit setting rate, thin peel, and rich flavor. Similarly, effective measures to minimize postharvest decay of “Shucuizao” fruits are the priority for researchers. In this study, the effects of exogenous melatonin on visual appearance and physicochemical changes of jujube fruit during cold storage were investigated. In particular, we provided an effective scheme to maintain physicochemical qualities of “Shucuizao” during cold storage.

## Materials & Methods

### Materials

Jujube fruits (*Ziziphus jujuba* Mill. cv. Shucuizao) were hand-harvested from an orchard in Wanfo Village (31°26′34″N, 104°46′18″E), Deyang City, Sichuan Province, with an altitude of about 660 m and a subtropical humid monsoon climate. At the reddening stage (commercial maturity), disease-free jujube fruits of uniform color and size were collected from the grafted seedlings (*Ziziphus acidojujuba* as rootstock) of five-year-old “Shucuizao” trees cultivated under a rain shelter. And the samples were brought back to the laboratory.

### Experimental design

The experiment was conducted at the Chengdu Campus of Sichuan Agricultural University (30°42′N, 103°51′E). 0.700 g of melatonin (BC grade; Sangon Biotech, Shanghai, China) was dissolved in 10 mL absolute alcohol (AR grade), followed by 7.5 L distilled water to form 400 µM solution. The five concentrations were as follows: 0 (distilled water, control), 50, 100, 200 and 400 µM, by adjusting the ratio of distilled water and 400 µM solution. The jujube fruits of the uniform maturity and size were screened and randomly divided into five groups, each containing 60 fruits and soaked in 4 L distilled water and melatonin solution with different concentrations for 2 h (Deng et al., 2021). After drying, the fruit were put into PE plastic bags (length 32 cm, width 45 cm, thickness 0.04 mm) with six round holes (diameter 1.5 cm) and placed at 0 °C for pre-cooling for 12 h. The fruits were then stored in an artificial climate chamber (ALH-358-3; Ningbo Jiangnan Instrument Factory, Zhejiang, China) under the following conditions: 0 ± 1 °C with 90 ± 5% relative humidity (Zhao et al., 2020). At storage times of 0, 3, 6, 9, 12 and 15 d, 10 jujube fruits were randomly selected for evaluation of appearance and textural changes and physicochemical changes analysis. Each treatment was completed with three replicates.

### Sample analysis

#### Evaluation of appearance and textural changes

The statistical methods for evaluating visual appearance followed Wu et al. (2015). The fruits were divided into four types as follows. Crisp fine fruits largely maintained their original crispness and firmness, had no disease spots, and had commercial characteristics. Crisp rotten fruits remained crisp and firm as a whole, but had varying degrees of disease and rot. Soft fruits were soft and diseased. Rotten fruits had visible disease spots, and loss of commercial value. The commercial value of the four grade fruits was from high to low was as follows: crisp fine fruits, crisp rotten fruits, soft fruits, and rotten fruits. The number of four grade of fruit was divided respectively by the total amount to get their respective percentages.

#### Determination of weight loss and firmness

Ten jujube fruits were weighed using a digital scale (Practum224-1CN; Sartorius, Göttingen, Germany). The weight loss was calculated by the following formula (Yang et al., 2021).

\begin{eqnarray*}\text{Weight loss} (\text{%})= \frac{mo-m1}{mo} \times 100 \end{eqnarray*}

*mo* is the initial weight and *m*1 is the weight measured during the storage period.

Fruit firmness was determined using a digital fruit firmness tester (GY-4; Jinyang, Beijing, China) fitted with a cylinder probe of 7.9 mm. Two sides of fruits along the equatorial region were chosen to measure firmness.

#### Concentrations of total soluble solids, titratable acidity and ascorbic acid analysis

Fruits including the skin were chopped up, mixed evenly, and the juice was collected to determine the total soluble solids (TSS) content using a portable digital refractometer (PAL-1; ATAGO, Tokyo, Japan). Sample (0.500 g) was extracted with distilled water, and the titratable acidity (TA) content (calculated as malic acid) was determined by the sodium hydroxide titration method (Zhang et al., 2012). Additionally, sample (0.400 g) was extracted with 2% oxalic acid, and the 2,6-dichlorophenol indophenol titration method was used to determine the ascorbic acid (AsA) concentration (Zhang et al., 2012).

#### Determination of superoxide dismutase activity and malonaldehyde content

The sample (0.500 g) was extracted with 10 mL phosphoric acid buffer (0.5 mM, pH 7.8), centrifuged (8000 rpm, 10 min), and the supernatant was used to determine the activity of superoxide dismutase (SOD) by the reduction method with nitrotetrazolium blue chloride (Giannopolitis & Ries, 1977).

The sample (0.500 g) was extracted with five mL trichloroacetic acid, and the supernatant was added with thiobarbituric acid after centrifugation and bathed in boiling water for 30 min. The absorbance of the extracts was determined at various wavelengths (450, 532, 600 nm) to calculated malonaldehyde (MDA) content (Sagi, Omarov & Lips, 1998).

### Statistical analysis

Statistical analyses in this study were conducted using the SPSS 20.0 statistical software (IBM, Chicago, IL, USA). Data were analyzed by one-way analysis of variance, with significant differences (Duncan’s multiple range test) assessed at the 5% confidence level. Pearson’s correlation analysis was carried out between visual appearance and physicochemical changes, and was used for heatmap analyses with Origin 2022 (OriginLab, Northampton, MA, USA).

## Results

### Effect of exogenous melatonin on visual appearance of postharvest jujube fruit

The various concentrations of exogenous melatonin had different effects on the percentages of crisp fine, crisp rotten, soft and rotten fruits during the storage period (Table 1). Compared with the control, 50, 100, and 200 µM melatonin significantly increased the percentage of crisp fine fruits after storage for 12 d and 15 d. In addition, the percentage of crisp fine fruits in the 50 µM treatment was approximately two and four times that in the 100 and 200 µM treatments at 15 d, respectively. The percentage of crisp rotten fruits in 50 µM melatonin treatment decreased by 75.18% (*p* < 0.05), 27.25% (*p* < 0.05), 27.35% (*p* < 0.05) and 24.51% (*p* < 0.05) at 3, 6, 9 and 15 d, respectively, in comparison with the control. Compared with the control, 200 and 400 µM melatonin increased the percentage of soft fruits at 15 d by 32.59% (*p* < 0.05) and 48.52% (*p* < 0.05), respectively, while there was no significant difference between 50 and 100 µM treatments. The percentage of rotten jujube fruits increased with storage time. Compared with the control, 100 µM treatments solution significantly reduced the percentage of rotten fruits by 20.26%, other concentrations, however, had little effect.

**Table 1:** Effect of exogenous melatonin on preservation of visual appearance of postharvest jujube fruit.

Index/%	Storage time/d	Control	50 µM	100 µM	200 µM	400 µM
Crisp fine fruits	3	66.9 ± 7.7bc	86.8 ± 11.5a	83.3 ± 7.0ab	82.9 ± 10.5ab	59.9 ± 7.4c
	6	49.9 ± 4.5b	66.9 ± 7.2a	56.6 ± 7.2ab	61.9 ± 4.5a	46.7 ± 6.6b
	9	46.7 ± 4.9ab	53.3 ± 6.2a	40.2 ± 6.1bc	30.8 ± 4.6c	20.2 ± 3.1d
	12	10.3 ± 2.0c	24.4 ± 2.8a	20.0 ± 3.6ab	16.7 ± 2.4b	10.1 ± 1.9c
	15	0.0 ± 0.0d	11.5 ± 1.8a	6.7 ± 0.8b	3.6 ± 0.7c	0.0 ± 0.0d
Crisp rotten fruits	3	26.6 ± 2.1b	6.6 ± 1.3d	10.2 ± 1.7cd	13.6 ± 1.5c	33.3 ± 3.1a
	6	36.7 ± 2.9a	26.7 ± 3.6b	30.1 ± 3.8b	27.7 ± 3.4b	41.3 ± 4.2a
	9	45.7 ± 5.0b	33.2 ± 2.9c	40.3 ± 4.7bc	48.4 ± 5.9b	65.5 ± 4.8a
	12	55.3 ± 5.2a	48.4 ± 4.8a	50.0 ± 4.3a	50.2 ± 6.0a	51.8 ± 7.5a
	15	50.6 ± 6.8a	38.2 ± 4.7b	50.8 ± 5.2a	38.3 ± 4.6b	40.0 ± 5.2b
Soft fruits	3	6.7 ± 0.6a	6.8 ± 0.7a	6.9 ± 0.5a	3.4 ± 0.4b	6.7 ± 0.6a
	6	10.3 ± 1.4b	6.6 ± 0.8c	13.3 ± 0.8a	10.4 ± 0.8b	10.2 ± 0.9b
	9	16.7 ± 1.5b	13.2 ± 1.1c	16.6 ± 0.9b	20.6 ± 1.8a	14.0 ± 1.3c
	12	20.6 ± 2.3b	17.4 ± 1.6b	20.3 ± 2.8b	16.8 ± 1.9b	25.8 ± 2.6a
	15	27.0 ± 2.1cd	30.8 ± 3.4bc	22.3 ± 3.9d	35.8 ± 3.5ab	40.1 ± 6.1a
Rotten fruits	3	0.0 ± 0.0a	0.0 ± 0.0a	0.0 ± 0.0a	0.0 ± 0.0a	0.0 ± 0.0a
	6	3.4 ± 0.4a	0.0 ± 0.0b	0.0 ± 0.0b	0.0 ± 0.0b	0.0 ± 0.0b
	9	3.4 ± 0.3a	0.0 ± 0.0b	3.6 ± 0.4a	0.0 ± 0.0b	0.0 ± 0.0b
	12	13.8 ± 1.2a	10.4 ± 1.3b	10.1 ± 1.1b	11.7 ± 1.0b	11.2 ± 1.4b
	15	23.2 ± 1.4a	19.3 ± 1.4ab	18.5 ± 2.1b	21.5 ± 2.3ab	20.2 ± 2.9ab

**Notes.**

Note: Values are means of three replicates ± SD and different lowercase letters in the same row indicate significant differences (Duncan’s multiple range test, *p* < 0.05).

### Effects of exogenous melatonin on the weight loss of postharvest jujube fruit

**Figure 1:**
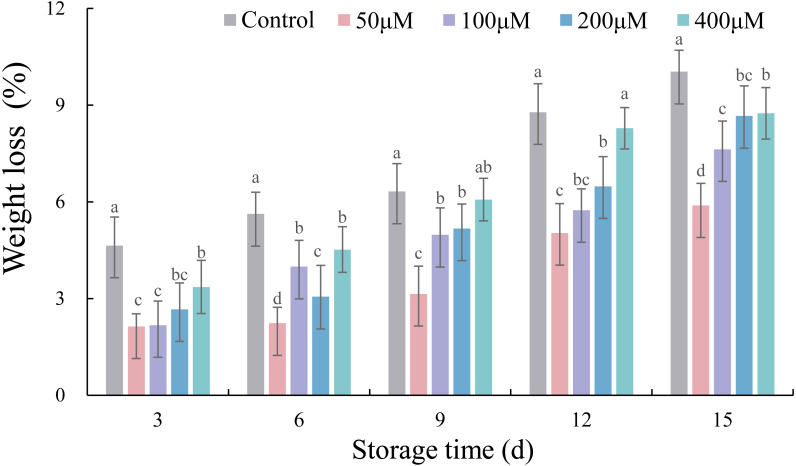
Effects of exogenous melatonin on the weight loss of postharvest jujube fruit. Values are means of three replicates ± SD and different lowercase letters at the same storage time indicate significant differences between treatments with different concentrations of melatonin (Duncan’s multiple range test, *p* < 0.05).

The weight loss of jujubes increased with the extension of storage time (Fig. 1). Except for 400 µM at 9 and 12 d, the different concentrations of exogenous melatonin significantly retarded the increase in weight loss of jujube fruit during storage compared with the control. The lowest weight loss during storage was in jujube fruits treated with 50 µM melatonin. Compared with the control, 50 µM melatonin reduced weight loss by 53.98% (*p* < 0.05), 60.21% (*p* < 0.05), 50.27% (*p* < 0.05), 42.65% (*p* < 0.05) and 41.29% (*p* < 0.05) at 3, 6, 9, 12 and 15 d, respectively.

### Effects of exogenous melatonin on firmness of postharvest jujube fruit

**Figure 2:**
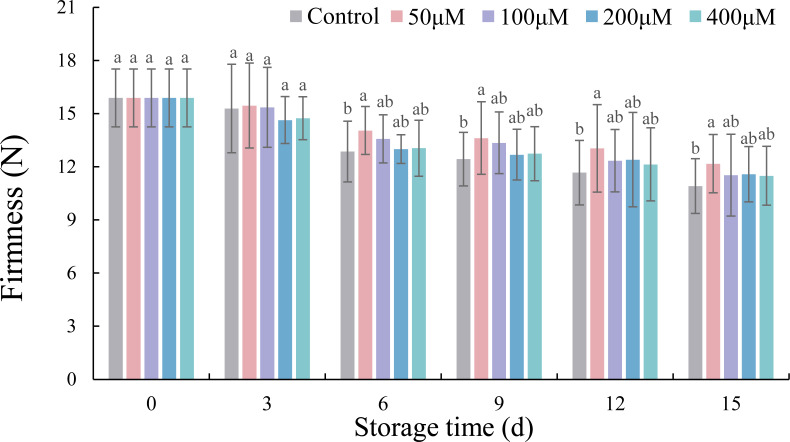
Effects of exogenous melatonin on the firmness of postharvest jujube fruit. Values are means of three replicates ± SD and different lowercase letters at the same storage time indicate significant differences between treatments with different concentrations of melatonin (Duncan’s multiple range test, *p* < 0.05).

The firmness of jujube fruit decreased with storage time and reached the lowest value at 15 d (Fig. 2). In comparison with the control, 50 µM melatonin increased the firmness of jujube fruit by 9.26% (*p* < 0.05), 9.56% (*p* < 0.05), 11.73% (*p* < 0.05) and 11.53% (*p* < 0.05) on 6, 9, 12, and 15 d, respectively. The other three concentrations of melatonin had no obvious effect on jujube fruit during storage.

### Effects of exogenous melatonin on TSS contents of postharvest jujube fruit

**Figure 3:**
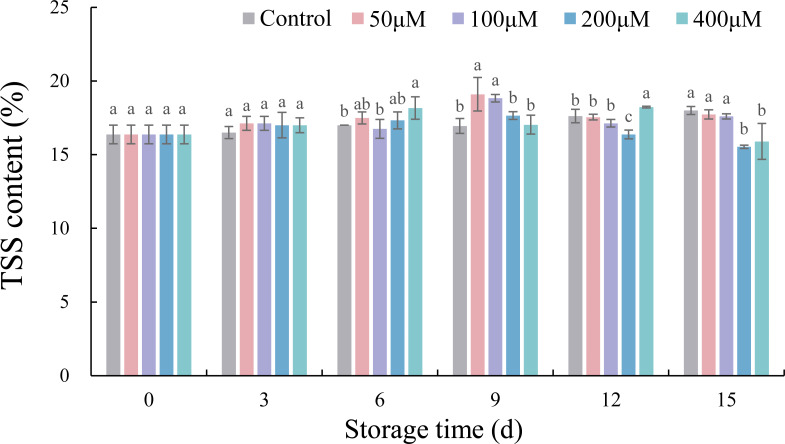
Effects of exogenous melatonin on TSS content of postharvest jujube fruit. Values are means of three replicates ± SD and different lowercase letters at the same storage time indicate significant differences between treatments with different concentrations of melatonin (Duncan’s multiple range test, *p* < 0.05).

The TSS contents of untreated jujubes gradually increased during storage, while the TSS contents of melatonin-soaked jujubes increased first and then decreased (Fig. 3). The range of change was 15.53%–19.10%. Compared with the control, 50 and 100 µM melatonin significantly increased the TSS contents on 9 d by 12.69% (*p* < 0.05) and 11.11% (*p* < 0.05), respectively. The TSS contents of fruits treated with 200 µM melatonin on 12 d were decreased by 7.09% (*p* < 0.05) in comparison with the control. Furthermore, fruits soaked in 200 and 400 µM melatonin solution significantly decreased the TSS content on 15 d by 13.70% (*p* < 0.05), and 10.34% (*p* < 0.05), respectively, compared with the control.

### Effects of exogenous melatonin on the TA contents of postharvest jujube fruit

**Figure 4:**
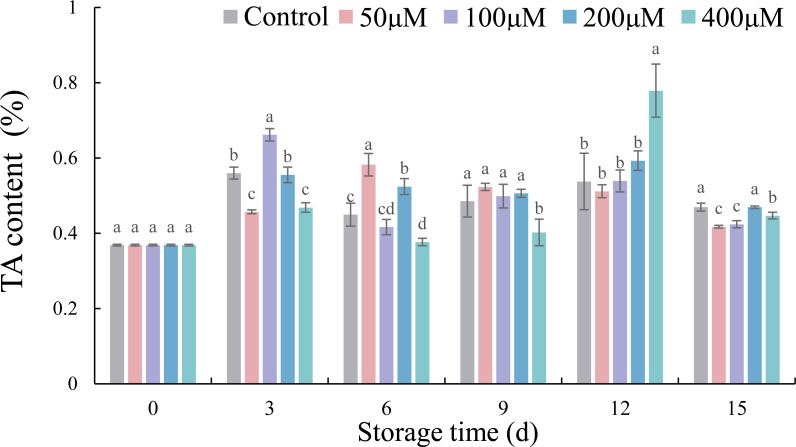
Effects of exogenous melatonin on TA content of postharvest jujube fruit. Values are means of three replicates ± SD and different lowercase letters at the same storage time indicate significant differences between treatments with different concentrations of melatonin (Duncan’s multiple range test, *p* < 0.05).

Except for 50 µM, changes in TA contents with melatonin treatments with progression storage interval followed the sequence of increase (0–3 d) then decrease (3–6 d), again increase (6–12 d) and finally decrease (12–15 d) (Fig. 4). Compared with 0 d, the TA content in each treatment increased to different degrees at 15 d. However, the fruit treated with 400 µM melatonin reached a peak value of 0.78%, which was a significant increase of 44.91% compared with the control, at 12 d. In addition, 50, 100 and 400 µM melatonin reduced the TA content of jujube fruits by 11.08% (*p* < 0.05), 9.71% (*p* < 0.05) and 4.85% (*p* < 0.05), respectively, after 15 d of cold storage, in comparison with the control.

### Effects of exogenous melatonin on the AsA contents of postharvest jujube fruit

**Figure 5:**
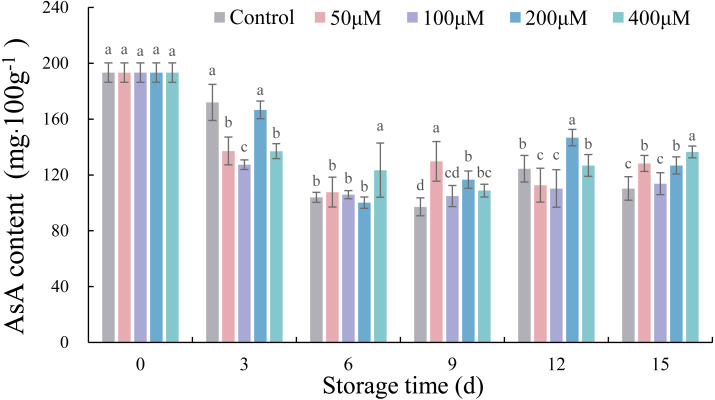
Effects of exogenous melatonin on AsA content of postharvest jujube fruit. Values are means of three replicates ± SD and different lowercase letters at the same storage time indicate significant differences between treatments with different concentrations of melatonin (Duncan’s multiple range test, *p* < 0.05).

The AsA contents of postharvest jujube fruit in each treatment decreased and then increased slowly during storage time, ranging from 97.17 to 193.36 mg g^−1^ FW (Fig. 5). After storage for 15 d, 50, 200 and 400 µM melatonin significantly increased the AsA contents of jujube fruit by 16.30% (*p* < 0.05), 14.98% (*p* < 0.05) and 23.80% (*p* < 0.05), respectively, in comparison with the control.

### Effects of exogenous melatonin on the SOD activity of postharvest jujube fruit

**Figure 6:**
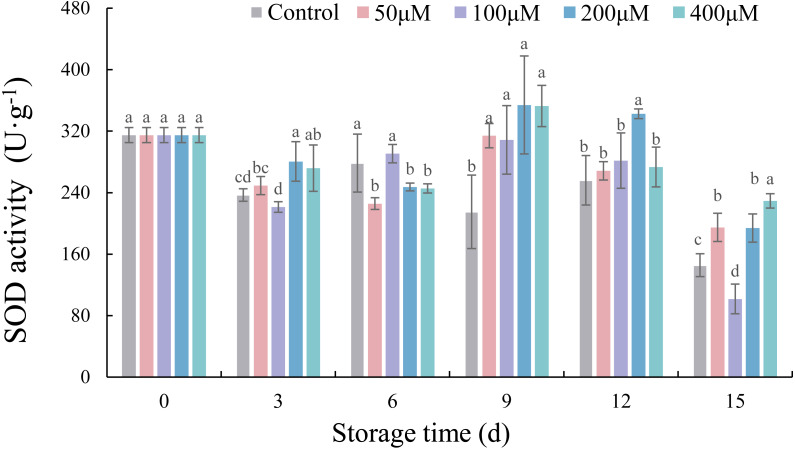
Effects of exogenous melatonin on SOD activity of postharvest jujube fruit. Values are means of three replicates ± SD and different lowercase letters at the same storage time indicate significant differences between treatments with different concentrations of melatonin (Duncan’s multiple range test, *p* < 0.05).

After the melatonin-soaking treatment, the SOD activity of jujube fruit showed a trend of decrease, increase, and then decrease during storage (Fig. 6). Compared with the control, the melatonin concentrations of 50, 100, 200, 400 µM increased the SOD activities of jujube fruits after storage for 9 d by 46.05% (*p* < 0.05), 43.47% (*p* < 0.05), 64.58% (*p* < 0.05) and 63.98% (*p* < 0.05), respectively. The SOD activities of all treatments reached the lowest values at 15 d, which ranging from 101.71 U g^−1^ to 229.33 U g^−1^. Compared with the control, 50, 200, 400 µM melatonin significantly increased the SOD activity value by 33.76% (*p* < 0.05), 33.17% (*p* < 0.05) and 57.43% (*p* < 0.05), respectively.

### Effects of exogenous melatonin on the MDA content of postharvest jujube fruit

**Figure 7:**
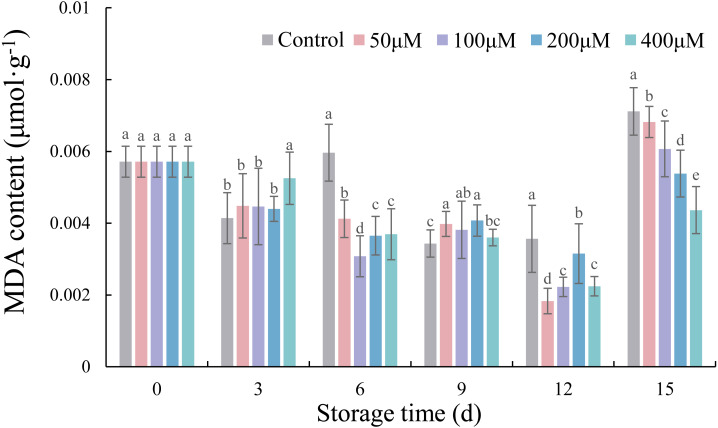
Effects of exogenous melatonin on MDA level of postharvest jujube fruit. Values are means of three replicates ± SD and different lowercase letters at the same storage time indicate significant differences between treatments with different concentrations of melatonin (Duncan’s multiple range test, *p* < 0.05).

The MDA content of melatonin-treated fruits decreased gradually from 0 to12 d of storage, but increased rapidly at 15 d (Fig. 7). Compared with the control, melatonin concentrations of 50, 100, 200, and 400 µM significantly reduced the MDA content of fruits on 15 d by 4.32% (*p* < 0.05), 14.71% (*p* < 0.05), 24.38% (*p* < 0.05) and 38.68% (*p* < 0.05), respectively.

### Correlation analysis between visual appearance and physicochemical changes

**Figure 8:**
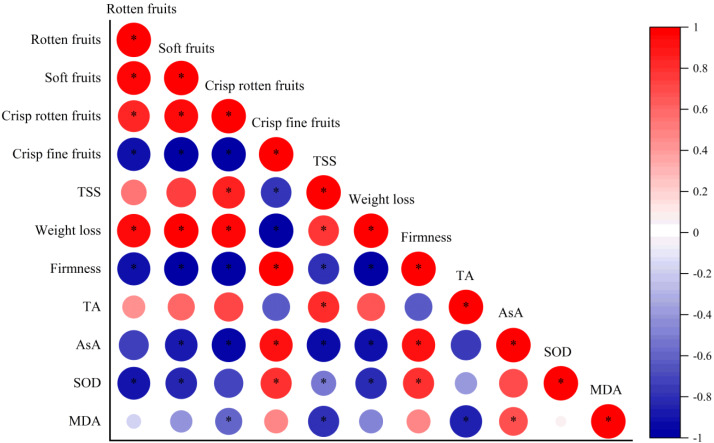
Correlation between visual appearance and physicochemical changes of postharvest jujube fruit as influenced by melatonin treatment. Colors from blue to red indicate that the correlation strength from negative to positive, and intense red color indicates that the more significant. An asterisk (*) indicates that a significant correlation at the 0.05 level.

The correlations between visual appearance and physicochemical changes of postharvest jujube fruit were diverse (Fig. 8). The percentage of crisp fine fruits was positive with firmness, AsA content and SOD activity, but negative with weight loss, TSS content and the percentage of rotten, soft and crisp rotten fruits. The percentage of crisp rotten fruits was positive with TSS content, weight loss and percentage of soft and rotten fruits, and was negative with firmness, contents of AsA and MDA. The percentage of soft fruits was positive with rotten fruits percentage and weight loss, but negative with firmness, AsA content and SOD activity. The percentage of rotten fruits was positive with weight loss, but negative with firmness and SOD activity.

## Discussion

The decrease of visual appearance during cold storage is closely related to weight loss and firmness, which severely affect taste and economic value of post-harvest fruits and vegetables (Fernández-León et al., 2013; Selahle, Sivakumar & Soundy, 2014). Some metabolic activities, such as transpiration and respiration, the main factors influencing the increase of weight loss and decrease of firmness with the extension of storage time, occurred in postharvest jujube fruits (Lufu, Ambaw & Opara, 2020; Park et al., 2015; Yang et al., 2021). Recently, studies on strawberry (Liu et al., 2018), pitaya (Ba et al., 2021), peach (Gao et al., 2016) and plum (Yan et al., 2022) showed that exogenous melatonin significantly inhibited the increases in decay and water loss, and the decline in firmness. In the present study, firmness had the strongest positive correlation with crisp fine fruits and negative correlation with crisp rotten, soft and rotten fruits, while weight loss was just the opposite. Thus it could be seen that weight loss and firmness were indicators of visual appearance. Moreover, jujube fruits immersed in 50 µM melatonin solution showed the best performance in delaying weight loss and firmness decline, thereby promoting the percentage of crisp fine fruits. The best melatonin concentration in this experiment was inconsistent with those reported in studies by Tang et al. (2020) and Deng et al. (2021). We speculated that differences in storage temperature and variety were the main reasons for the difference.

As the most vital intrinsic qualities affecting flavor of fruit, the contents of TSS and TA varied during cold storage (Zhang et al., 2012). Studies showed that exogenous melatonin could maintain the postharvest quality of sweet cherry (Miranda et al., 2020; Wang et al., 2019) and orange fruits (Ma et al., 2021). In this experiment, exogenous melatonin at concentrations of 50 and 400 µM inhibited the increase in TSS or TA contents at the end of storage, which was similar to studies on plum (Bal, 2019), mango (Liu et al., 2020) and jujube (Wang et al., 2021a; Wang et al., 2021b). There was a positive correlation between TSS content and weight loss in jujube fruits, which speculated that the increase of TSS content might be related to water evaporation during cold storage (Reche et al., 2019). The results showed that an appropriate concentration of exogenous melatonin could effectively maintain the desirable flavor of jujubes under cold temperature storage (Wang et al., 2021a; Wang et al., 2021b).

AsA is not only an important nutritional quality character of jujube fruit, but also part of non-enzymatic antioxidative defense system (Zhang, Huang & Li, 2016). In this study, exogenous melatonin of 50, 200 and 400 µM delayed the decline of AsA content in postharvest fruits at 15 d. As seen by correlation analysis, high level of AsA content was correlated with decline in weight loss and increase in firmness of jujube fruits treated with melatonin, thereby indirectly affect visual appearance. Similarly, application of exogenous melatonin resulted in higher AsA contents in plum (Bal, 2019), peach (Gao et al., 2016), pitaya (Ba et al., 2021) and tomato (Liu et al., 2016). It was speculated that the decrease in the AsA content may be related to triggering of ASA-GSH cycle to enhance the cold tolerance of jujube fruit (Miranda et al., 2020). Our results suggested that melatonin at appropriate concentrations enhances antioxidant activity and maintains the quality of postharvest jujube fruits (Tang et al., 2020).

**Figure 9:**
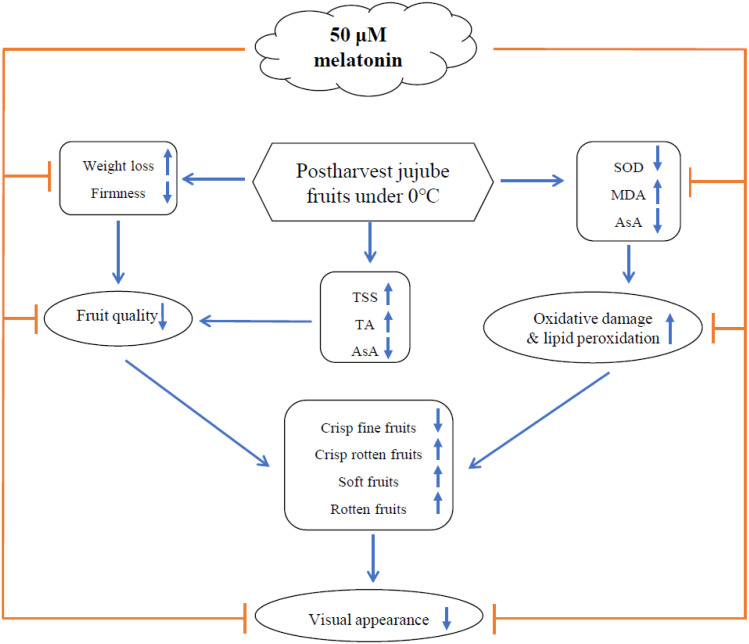
Proposed model regarding of the 50 µM melatonin on the visual appearance and physicochemical changes of postharvest jujube fruits under 0 °C.

It is well known that the negative effects of low temperatures in plants are oxidative damage and lipid peroxidation (Valenzuela et al., 2017). Exogenous melatonin reduced the MDA contents of postharvest apples (Onik et al., 2021), pitaya (Ba et al., 2021) and litchi (Zhang et al., 2018), while enhancing SOD activities, which contributed to delaying senescence by improving cold resistance during storage. In this study, jujube fruits soaked with 50, 100 and 400 µM melatonin increased SOD activity and decreased MDA content on 15 d, compared with the control. Combined with the AsA contents, the results showed that melatonin treatment reduced the oxidative stress induced by low temperature through the active oxygen scavenging system and AsA-GSH cycle, thereby enhancing the cold resistance of jujube fruits, which was similar to results in peach (Gao et al., 2016). Correlation analysis also suggested that the melatonin treatment improved the rate of crisp fine fruits by increasing of antioxidant capacity.

## Conclusions

In summary, based on the comprehensive evaluation of physicochemical qualities, we concluded that 50 µM melatonin improved the visual appearance of jujube fruits, and maintained quality changes, which may be attributed to its capacity to mediate antioxidative action (Fig. 9). Therefore, we proposed that exogenous melatonin treatment is a promising approach to preserve postharvest jujube fruits under 0 °C.

##  Supplemental Information

10.7717/peerj.14155/supp-1 Supplemental Information 1 Raw data

## Additional Information and Declarations

### Data Availability

The following information was supplied regarding data availability: The raw data is available as a Supplemental File.
